# Supplementary figures and images for: Biological action at a distance: Correlated pattern formation in adjacent tessellation domains without communication
Source: PLoS Comput Biol. 2022 Mar 28;18(3):e1009963. doi: 10.1371/journal.pcbi.1009963 (PMC8989308; doi:10.1371/journal.pcbi.1009963)

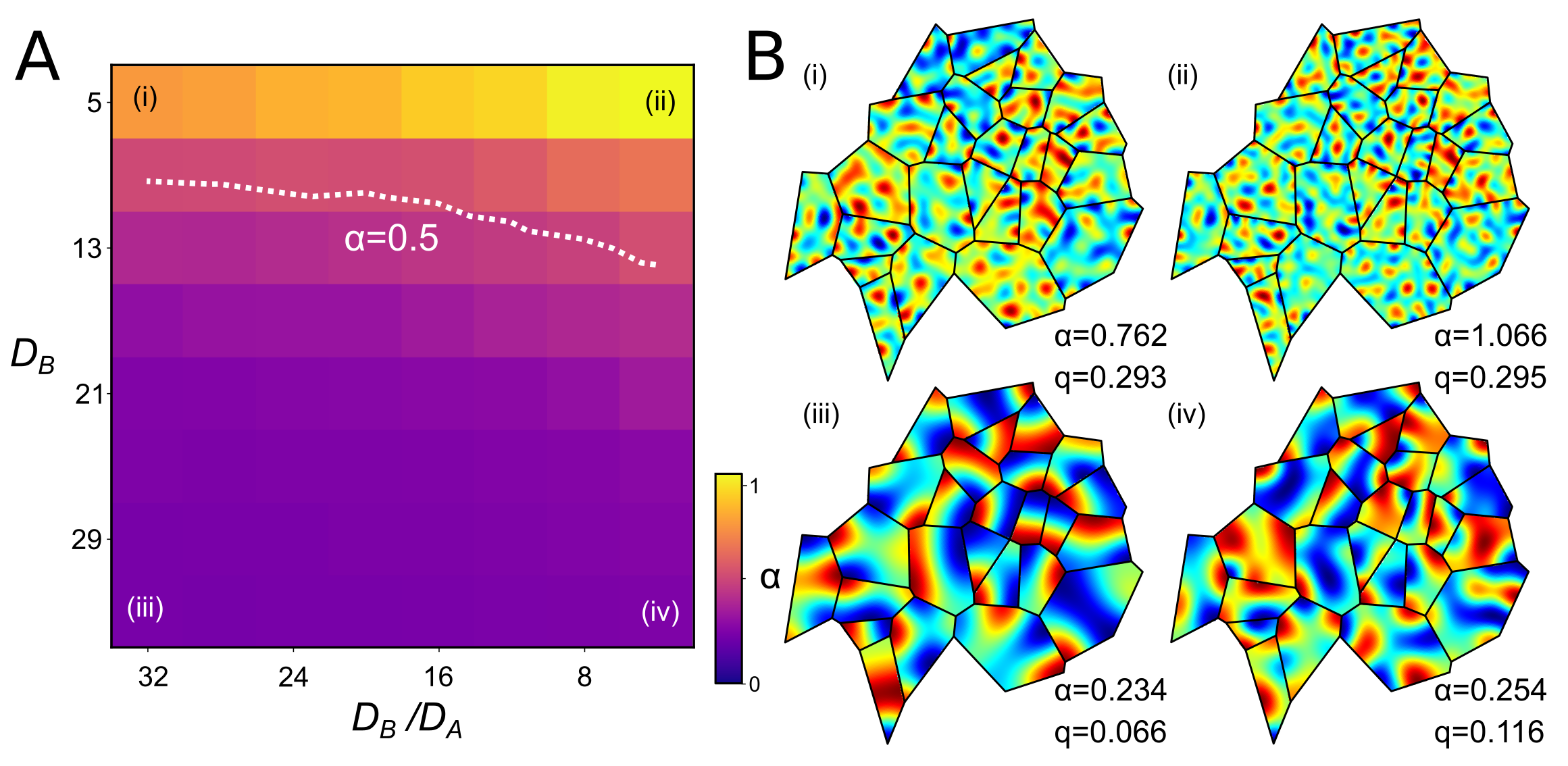

Supplement: S1 Fig — We conducted a parameter sweep comparable to that presented in Fig 5, instead using the Schnackenberg reaction-diffusion system [22], whereby patterns form via the Turing mechanism. This system has no nonlinear diffusion term and its dynamics are driven by the magnitudes and ratios of two parameters that scale the linear diffusion operators, DA and DB (see [23]). A shows values of α obtained across a large portion of the parameter space, with the ratio of the two diffusion parameters, DB/DA, decreasing along the horizontal axis. The gradient in α corresponds well to that shown in Fig 5 using the Keller-Segel model. B shows examples of the field patterns for parameter values corresponding to the four corners in A. (TIF) [file pcbi.1009963.s001.tif]
