# Supplementary material for: Biological action at a distance: Correlated pattern formation in adjacent tessellation domains without communication
Source: PLoS Comput Biol. 2022 Mar 28;18(3):e1009963. doi: 10.1371/journal.pcbi.1009963 (PMC8989308; doi:10.1371/journal.pcbi.1009963)
Supplement: Attachment — Submitted filename: Brooke2021ResponseToReviewers.pdf [file pcbi.1009963.s002.pdf]

Response to Reviewers: Manuscript PCOMPBIOL-D-21-01415

Dear Professor Maini and Professor Beard,

Thank you for the opportunity to revise our manuscript. We are pleased that the comments provided by the two reviewers are generally positive, and we found the points raised by both to be constructive and helpful, and to reflect a high level of engagement with the material, for which we are grateful. In response to the points raised the major changes that we have made to the manuscript include:

- Two new figures which help to more clearly motivate (Fig 1) and explain (Fig 2) the basic result of the paper, and a new panel (Fig 4A) demonstrating the robustness of our analyses.
- Substantial re-organisation of the Results section to include additional analyses requested, in each case each adding support to the original conclusions
- Significant refinement of the final Discussion section to keep the discussion more focussed on the immediate implications of the results
- Inclusion of additional references to further support our conclusions

Please find below a point-by-point response to each of the comments provided by the two reviewers. The original reviewer comments are reproduced in full below in blue, and our responses are in black. We hope that you will agree that the paper is stronger as a result of the modifications.

Sincerely,

John Brooke, Seb James, Alejandro Jiminez-Rodriguez, and Stuart Wilson

Reviewer #1:

In this work, the authors are interested in understanding the interplay between boundary conditions and pattern formation on tessellated domains. They simulate reaction-diffusion equations on a range of tessellated domains, including collections of equilateral triangles, isosceles triangles, Voronoi cells, and Voronoi cells with rounded edges (assuming that pattern formation is independent in each cell). The authors propose a new means of elucidating whether or not domain boundaries actively constrained pattern formation by comparing the patterns that emerge across the tiles of the tessellation. They focus on the patterns with long wavelengths near the tile edges and show that the patterns sampled from adjacent tiles are correlated according to an overall bimodal distribution. It's an interesting, useful idea to compare the alignment of patterns in different regions, and the authors highlight how this could show up in biological applications. I have some questions about how the authors chose thresholds rigorously and how their results are related to patterns with small wavelengths. The Discussion is very broad; I recommend including more figures and more biological motivation, and reducing the Discussion breadth to better focus on the manuscript's contribution.

Our thanks to reviewer 1 for the thorough and insightful comments.

Main comments:
(1) I recommend moving the PDEs from Methods to the start of the Results section and discussing what the terms and notation in them means there.

We have now moved the partial differential equations to the beginning of the Results section as suggested. Please note that rather than highlight the whole section in blue in the track-changes version of the revised manuscript, we have used blue only to highlight the edits to the original text that have been used to incorporate the surrounding text into its new location in the paper.

(2) At the bottom of page 7 and beginning of page 8, I do not follow how the choice of the two alpha thresholds (alpha = 0.5 and alpha = 0.75) are made rigorously. Is this based on a single set of simulation conditions, for large wavelength patterns? I think it would be more meaningful if, for example, the authors repeated this study (constrained and control) across many more parameters, computed the resulting alignment distributions for all these cases, and then clustered the resulting distributions into two groups in an unsupervised way. If the resulting groups broke into constrained and control and held up the alpha thresholds, that would be stronger.

We have addressed this point in two ways.

First, we have moved our consideration of Equation 2 from the fourth to the third Results subsection, to help the reader appreciate earlier on that the threshold value alpha=0.5 derives from the analysis of the beta distribution that is developed in full in the Methods section, rather than from simulation results. We have also included a subheading in the Methods "Derivation of a test for the influence of boundary shape" to further clarify that the 0.5 threshold was obtained by analysis not simulation.

Second, as suggested by the reviewer, we ran a new parameter sweep through the full range used for that reported originally, but now also for solutions obtained in the control condition, where the boundary shapes were not a constraint on pattern formation (the boundaries were circular). We discovered that after a simple log transformation to the datapoints, the alpha values were very clearly separable between the two conditions, across the full range of parameters. As explained in two new paragraphs at the top of page 8 we trained a simple perceptron classifier to discriminate the two classes of data, and we added a new plot of those data (Fig. 4A) that also shows the perceptron decision boundary and confirms the clear separation between conditions. As now explained on page 8 the more practical threshold value (alpha=0.75) was obtained from inspection of the numerical solutions in the control condition, using parameters for self-organisation that gave the lowest, and thus the *most conservative estimate* of the bimodality induced by boundary constraints (D_n=36, chi=0).

(3) In Figures 1 and 3, could the authors provide more intuition about how the reader should interpret the distribution for random edge pairs? Should I think of this as similar to just capturing alignment between patterns and boundaries? Could the authors test whether the distributions for adjacent edge pairs and random edge pairs are significantly different?

The main reason to include the analysis of randomly matched edges is to show that the distribution of correlations can be bimodal even without sampling from adjacent edges, due to the alignment between the patterns and the boundaries (as noted by the reviewer), as is the case with equilateral triangles (where the adjacency of domains by their layout on a tessellation is arbitrary), and that for shapes that tessellate in a more specific way (e.g., isosceles and scalene triangles) the bimodality is exaggerated. This point is clarified by the inclusion of the new figure 2, and its caption. Our analyses and conclusions do not require for the distributions for adjacent and random pairs to be statistically different (bimodality is evidence that boundary shape constrained pattern formation even in the equilateral triangles case where the distributions are identical). However, for completeness, we have now included the results of statistical tests (Kolmogorov-Smirnoff tests between adjacent and randomly matched edge pairs) as requested, at the end of pages 5, 6 and 8.

(4) The paper would benefit from more figures. The discussions around line 62 and line 70 in the Introduction could include figures (e.g., at line 70, an image of the rodent cortex, with barrels and sub-barrel patterns highlighted). I recommend including an illustration for the discussion on page 4 about type 1, 2, and 3 edges. In the discussion around line 145, I recommend referencing Figure 1.

The revision now includes 2 entirely new figures and a new panel in Figure 4. New Figure 1 includes images of the rodent cortex, with barrels (Fig 1B) and subbarrels (Fig 1C) highlighted. Figure 2 now supplements the discussion of the different edge types in the equilateral versus isosceles cases, and we think the examples developed in the main text are now much clearer as a result. We have also included additional references to Figure 2 and Figure 3 (originally Figure 1) at the appropriate points.

(5) The language throughout is wordy (passive voice, long sentences, broad in the Discussion), and this can make it hard to understand the authors' points. The breadth of the Discussion did not seem to match the results in the manuscript; my understanding of the main results of the paper are that the authors proposed a way of identifying the impact of boundaries on pattern formation in tessellated domains by comparing the patterns in adjacent tiles. The Discussion was much broader, and I would recommend reducing this. For example, the sentence "Thus the symmetry breaking by which pattern forms in the short term gives rise to symmetries that persist in the longer term" at line 437 did not seem to match the main results and contribution of the paper.

We have substantially reduced the original discussion, focussing on the final part (the broader discussion of constraint closure), and reducing it by 340 words and from three paragraphs to one. As a result, the discussion is much more focussed on topics that are directly relevant in light of the specific results of this paper. We have retained some discussion of constraint closure, because this is the concept that we used to motivate the study in the Introduction, albeit with a narrower focus. Note that the specific sentence referred to has been removed as a consequence of these edits. Edits made in response to numerous sentence-level comments helpfully provided by reviewer 1 below have made the revised paper more readable throughout.

(6) The authors discuss several applications in the Discussion, and I would suggest moving some of these applications into the Introduction instead. This would help motivate the manuscript and provide concreteness. For example, at line 48 in the Introduction, when the authors discuss shape emerging subsequently to, simultaneously with, or independent of pattern formation, it would be useful to include some references and example biological applications for each of these options.

We have addressed this point in part by the inclusion of the new Figure 1, and its caption, which includes an image of the giraffe patterning both as an extra example of a naturally occurring Voronoi tessellation and to preface the extended discussion of this specific application in the later Discussion. This new Figure adds to the concreteness of the Introduction. We have not edited the sentence that was originally on line 48. The point we are making here is that it is difficult to discriminate between these cases based on current methods of analysis, hence providing concrete examples of each case is only possible once the analysis we report in the paper has been developed.

(7) At line 470, could the authors clarify what they mean by x_1 (for example, through a figure)? What does it mean that they are using the grid points along and just inside the boundary vertex? How far inside? At line 476, does the authors' choice to increase the longer vector by linear interpolation make sense when the pattern has a shorter wavelength (as in Fig. 4Bii)? It would be helpful if the authors could discuss this situation here.

Addressing the first half of this comment, we have clarified this point in the revised Methods section, where we now refer to the 'hexagon-to-hexagon distance' and then state "Vectors $\mathbf{x}_1$ and $\mathbf{x}_2$ each contained the solution values in the hexagonal grid points along (and one hexagon-to-hexagon distance inside) a boundary vertex from either domain. The boundary lines are shown in black in Figs. 3, 4, 5, and the samples $\mathbf{x}_i$ were taken immediately adjacent to these boundary lines on either side.". (This is also underscored in the new paragraph at the end of page 5).

Addressing the second half of this comment, we have also included in the revised Methods “When comparing randomly matched edge vectors, the length of the shorter vector was increased to match that of the longer vector by linear interpolation. For all such results, the distances between interpolation points, even for the shorter vectors, were far smaller than the wavelengths that were observed to be amplified by pattern formation. Interpolating along the shorter vector was therefore appropriate, and preferable to downsampling along the longer vector, to avoid discarding information.”

Minor comments and typos:
(1) I recommend rewriting the sentence at line 30 (the last sentence in the Author Summary); it was unclear.

We have revised this line to: “Our analysis provides a means for testing current theories about the fundamental role that constraints play in organising biological systems.”

(2) I do not think the phrase “That is,…” at line 98 is a sentence.

We have revised to: “Specifically, correlations are to be expected if the boundaries of adjacent domains abut, such that the domain shapes constitute a tessellation.”

(3) Around line 114, is there a reference for the discussion about where the reaction collects in the triangles? Additionally, related to one of my main comments, it would be clearer if the equations were introduced here rather than in Materials and Methods. Then the authors could discuss what “reaction collecting in the corners of the triangle” means in terms of the values of eta and c.

We have retained from the text of the original submission “And it is known that patterns formed by reaction-diffusion systems tend to be strongly influenced by the presence of definite angular intersections at the vertices [19].”, where reference 19 is an excellent study of exactly this point:

[19] Jung CY, Park E, Temam R. Boundary layer analysis of nonlinear reaction–diffusion equations in a polygonal domain. Nonlinear Analysis: Theory, Methods & Applications. 2017;148:161–202. doi:https://doi.org/10.1016/j.na.2016.09.018.

We have moved the PDE equations to the start of the Results section as suggested, and referred to n and c in the corresponding text, which now immediately follows that equation.

(4) I am not familiar with the arrow notation the authors use in the Results section (p_a ->).

We have added a note to clarify that the arrow symbol denotes ‘tends to’.

(5) At line 149, should this be “with the bases’ perpendicular…”?

This typo has been fixed.

(6) At line 182, should it be “Fig 1D” rather than “(Fig 1D)”?

This typo has been fixed.

(7) At line 185, should it be “Dirichlet form” rather than “Dirichletform”?

We have now enclosed the term Dirichletiform (we’d missed out the adjoining i in the original submission) in quotation marks and provided a reference to Senft & Woolsey (1991) who used this term in a similar context.

(8) At line 188, I think it should be “To establish whether” rather than “So to establish”.

We have revised this phrase to be “Therefore, to establish…”.

(9) In the paragraph at line 200, it would be helpful if the authors could reference a figure showing the sub-barrel patterns and provide more biological background for readers who are not familiar with this application.

In response to major point 4 of reviewer 1 above we have included the new Figure 1, which includes the sub-barrel patterns (Fig 1C), as well as a description in the caption that provides more biological background, and we have referred to that figure at the suggested point (corresponding to original line 200) in the text. We have also now qualified that the patterns relate to “thalamocortical innervation density” and referred back to the Introduction, where the biological background has been provided.

(10) At line 228, can the authors include how many images they analyzed?

We have now clarified this with an edit in the second paragraph of the corresponding section.

(11) At line 260, the term beta(alpha, beta) is unclear. Could the authors choose a different variable name for the second beta?

Thank you for spotting this mistake. The first beta has now been capitalised, as per convention.

(12) At line 290, there is a space missing at “Fig.2”.

This typo has been fixed.

(13) The sentence starting “By showing that” at line 330 in the Discussion is long, and I would recommend rewriting. As it is, it read to me like the main hypothesis was that patterns form by reaction-diffusion, but I think the main hypothesis is that domain boundaries actively constrain pattern formation by reaction-diffusion.

We have simplified this sentence to:

“Our results show that the effect is not to be expected in tessellated domains whose boundaries did not constrain pattern formation (Fig. 3B). Hence they establish how bimodality in the distribution of correlations measured across adjacent edges of a tessellation (and specifically the presence of anti-correlations) can be used to test the hypothesis that the domain boundaries constrained pattern formation via reaction-diffusion.”

(14) At line 350, could the authors provide a reference and more biological background?

In this paragraph we explain how the alignment of patterns could influence the transport of materials between domains in the case where dense patterning would correspond with the presence of obstacles to the transport of materials. This is a hypothetical example, and one which we feel is explained in a logically complete, correct, and uncontroversial way, and hence one that does not require a supporting reference to the work of others. We note that the following paragraph (beginning “As an interesting example...”) develops a concrete biological example (the constraints of Giraffe skin patterning on blood supply for thermoregulation), which illustrates the point in some detail, and which includes two supporting references. So we hope that on a re-read the reviewer will agree that changes are not necessary here. Inclusion of new Fig. 1A should better prepare the reader to consider this discussion point.

(15) At line 390, the sentence “The relationship between these two patterns…” is long and difficult to parse.

We have split this sentence into two and simplified to:

“The relationship between these two patterns that is suggested by the present results provides a potential geometrical basis for the integration of sensory information. The alignment of within-barrel and between-barrel maps could render downstream cells sensitive to the coherence between single-whisker deflection directions and multi-whisker deflection intervals resulting from movement of tactile stimuli through the whisker field [34].”

(16) At line 426, could the authors add the reference for the quote?

The quote is from reference 2 (Montevil et al., 2016; p42). However, in addressing the other comments, this quote has been removed from the revision.

(17) At line 464, consistency-wise, the authors use “Eqs. 2”, but “Eq. 2” is used in all other places in the manuscript.

We have revised this reference to ‘Eq. 2’ for consistency.

(18) In Materials and Methods, are r and C the same thing? Additionally, cosine is sometimes italicized and sometimes not throughout the equations on pg. 23.

We have simplified the description of variables in the Methods section and as a result modified all references to C (originally used as an intermediate variable in the derivation) to r for clarity. We have also fixed the formatting of the cosine (using latex $\cos$) throughout.

(19) At line 496, could the authors rewrite this line (as is, it says that the distribution of correlations is cos(theta), but cos(theta) is not a distribution)?

We have edited this line to “Therefore the distribution of correlations consists of values given by $\cos (\phi)$...”

(20) At line 497, I did not follow why “over [0,pi] or over [pi,0]” is needed; can this just be “over [0,pi]”?

This was a hangover from an initial draft of the section. We’ve now corrected to $[-\pi,\pi]$.

(21) In reference [1], an accent character is missing in Montevil’s name.

This typo has been fixed.

Reviewer #2: In this paper, the authors continue their study of patterns in the whisker barrel system of the rodent. Their somewhat flashy title refers to the conjecture that if two domains have the same kinetics and diffusivities, then the patterns that appear via a Turing type instability will be similar and highly correlated along the edges even though there is no direct coupling. I think this is what they mean by "action at a distance." There are a few points I would like them to address.

Thank you to reviewer 2 for the helpful suggestions and pointers to additional literature.

First -- there are similar phenomena in the temporal domain where two uncoupled limit cycles are subjected to correlated fluctuations and thus begin to synchronize. This is called the "Moran effect"

and also "stochastic synchronization". I suggest that the present phenomena is closely related at least in a qualitative sense and they might want to discuss this literature.

Thanks for pointing us to the Moran effect, which we agree is related in an interesting way to the effect we have established here. We have included a paragraph in the Discussion as follows, with appropriate references:

“Ours is not the first demonstration that biological variables can become synchronised in spatially separate populations that do not communicate directly. For example, it is well known that the similarity in weather patterns between two locations tends to decrease with the distance between them. And as such, population dynamics in two separate groups of conspecifics that do not interact will tend to be correlated if their habitats are nearby, due to the effects of a common weather pattern as a mediating ‘third’ variable. This is an example of the “Moran effect” [21, 22, 23, 24]. The effect we have described is distinct in two ways. First, it arises from comparisons between the structures of patterns that vary in space and time (i.e., in systems described by partial differential equations), whereas the Moran effect describes temporal fluctuations only and is thus typically modelled using systems of ordinary differential equations. Secondly, the correlations studied here originate from the constant effect of the boundary conditions on pattern formation, rather than by the common influence of any time-varying quantity. We expect that both effects may yet be understood as instances of a more general class of phenomena by which spatial relationships between environmental variables (i.e., in terms of their proximity or boundary shapes) induce correlations between otherwise decoupled populations. To this end, future investigations may extend the analysis developed here to the study of systems whose solutions oscillate in both space and time.“

Secondly -- it is my impression that in large domains (or in the context of the paper, when the diffusivities and chemotaxis parameters are reduced, there can be many final patterns depending on the initial data. While they did address this in Fig 4, I remain skeptical that the correlations will be high when the pattern becomes more granular.

In addressing point 2 of reviewer 1, we conducted an additional parameter sweep, which again includes regions of parameter space that yield high spatial-frequency patterns, this time in the control condition where boundary shape did not influence pattern formation. We report in the revision a clear difference in alpha, the coefficient of the beta distribution that essentially measures the bimodality of the correlations, between the two conditions and across the full range of parameters. As shown in the new Fig 4A, the distinction between alpha in the two conditions reduces as the patterns become more complex, but it nevertheless remains across the full range, as evidenced by a decision boundary that can be drawn between the two populations. We agree that this is quite a remarkable finding, and trust that the new figure will help to alleviate the reviewer’s initial skepticism (Note that Fig. 4 in the original is now Fig. 5).

Third--what if there are small differences in the parameters in each of the barrels? How sensitive if the correlation structure to such heterogeneities which i would imagine are pretty common in biological systems.

We found that the numerical results are highly robust to the parameters. We ran additional simulations and in the revision we have added the following paragraph:

“It is possible that amongst the domains of a biological tesselation the control parameters for self-organisation may show some variation. To determine the robustness of the reported effects we therefore re-ran simulations with parameters in the mid-range of the space that was tiled by our initial parameter sweep ($D_n = 6.0$, $D_c = 0.3D_n$, $\chi = 6.0$) and then randomly perturbed these values in each domain by up to 10%. Compared to the unperturbed case, distributions of (adjacent) correlations were

not statistically different (Kolmogorov-Smirnov test, p = 0.997). When the control parameters were perturbed by up to 50% in each domain, the correlations appeared to diverge a little, but not enough to reject the null hypothesis that they were drawn from the same distribution (p = 0.31).”

Other than these comments, I enjoyed this paper. They is an extra "are" in line 22.

Thank you. This typo has now been fixed.
