# Supplementary material for: Biological action at a distance: Correlated pattern formation in adjacent tessellation domains without communication
Source: PLoS Comput Biol. 2022 Mar 28;18(3):e1009963. doi: 10.1371/journal.pcbi.1009963 (PMC8989308; doi:10.1371/journal.pcbi.1009963)
Supplement: Attachment — Submitted filename: ResponseToReviewers.pdf [file pcbi.1009963.s003.pdf]

Re: PCOMPBIOL-D-21-01415R1

Dear Professors Maini and Beard,

Thank you very much for the opportunity to submit a revised version of our paper ‘Biological action at a distance: Correlated pattern formation in adjacent tessellation domains without communication’ in response to the comments of the two reviewers. Please find below our responses to the reviewer comments.

Sincerely,

John Brooke, Sebastian James, Alejandro Jiminez-Rodriguez, Stuart Wilson

Reviewer #1:

Thank you to the authors for their careful revisions and thoughtful responses. I recommend acceptance.

Minor comments:
(1) Figure 1: Perhaps “Skin of giraffes” or “skin samples of giraffes” rather than “skins of giraffes”
(2) Figure 2A: I think the semicolon should be a comma: “patterns emerge, with extreme” rather than “patterns emerge; with extreme”.

Thank you very much to Reviewer 1. We have made the two suggested edits.

Reviewer #2:

The authors addressed all of my concerns, but i saw a comment in the paper on page 4 that had somehow escaped my earlier reading. "most of the simulations reported we will set $\chi = 0$," This makes me wonder how the authors got their numerical results. If chi=0, then in equation (1), n(x,t) -> 1 (assuming no-flux boundaries) since this is a then just a linear PDE and the solution can be exactly written down. In that case, f(n) -> constant and c(x,t)-> constant so that there will never be any pattern formation and thus there will be no patterns on the edge. If this statement is not true than i am happy to accept the paper, but if indeed chi=0, then the authors have not simulated the equations (1) since with chi=0, there can be no patterns

Reviewer 2 is absolutely correct. And we are a little embarrassed to have missed this point previously! Addressing this has revealed the effects that we reported, and the conclusions that we have drawn, to be stronger than originally presented.

The reviewer is correct that spatially inhomogeneous patterns should not be stable in the Keller-Segel system when the non-linear coupling term (χ) is smaller than the diffusion constant (D_n), as explained clearly in the paper that we cited by Ermentrout and colleagues (2009; reference 12). We missed this previously because of an issue with how we were checking that the magnitudes of the fields had converged, and our code was (under certain circumstances) erroneously returning the current state of the system as the converged solution rather than recognising these non-converged fields to be transient. Hence the patterns that we analysed originally for χ=0 represented transient unstable solutions that were in fact still decaying towards the spatially homogeneous state, i.e., to the absence

of any spatial patterning. After careful investigation we fixed the bug by refining the halting condition in our numerical integration algorithm, and we have verified that further integration when $\chi<D_n$ does eventually lead to the homogeneity predicted by Reviewer 2.

Integrating until the a more robust convergence criterion has been met also yields longer integration periods in some other regions of the parameter space, so we have now re-run all simulations with this criterion (excluding the $\chi<D_n$ region), and updated our report of the outcome of all of the relevant statistical tests in the paper. The resulting simulation data support our original analysis and conclusions more strongly. For example, excluding the region $\chi<D_n$ now reveals that the α metric varies smoothly with the χ and D_n parameters across the full parameter space (revised Figure 5), and this metric is now shown to fall below the analytically derived α=0.5 criterion across an even wider range of the parameter space. Similarly, the separation between α values obtained between the control and boundary-constrained conditions (revised Figure 4) has become even more strongly exaggerated. (Note that as such, the value of α estimated from this experiment to discriminate between the two cases is found to be essentially the same as that which we derived analytically, and so for clarity we have removed the short paragraph that previously referred to a separate 'numerically derived' α criterion in the revision.)

We had mistakenly been concentrating on χ=0 solutions in order to present what we had thought to be the conceptually simplest case. In the revision we now concentrate on $\chi=D_n$ instead (e.g., we have re-constructed Figure 3 using data from $\chi=D_n$ simulations). This raises a potential follow-up question about whether our predictions should hold for different pattern-forming systems. To confirm that they do, we have also now conducted a full parameter sweep using the simpler Schnakenberg reaction-diffusion system (see additional references 22 and 23), which does not include a non-linear diffusion coupling term like that which is parameterized by χ in the Keller-Segel model. The results are included in a new supplementary figure, which demonstrates that the alignment effects are equally strong for this alternative pattern-forming system, and thus further supports the generality of the predictions and conclusions of the paper.

We are very grateful to Reviewer 2 for spotting our error, and we are very glad to have had this opportunity to correct the mistake. We trust that the Editors will agree that the paper is even stronger as a result, and we look forward to your decision about its suitability for publication in *PLoS Computational Biology*.
